# Culture-Modified Bone Marrow Cells Attenuate Cardiac and Renal Injury in a Chronic Kidney Disease Rat Model via a Novel Antifibrotic Mechanism

**DOI:** 10.1371/journal.pone.0009543

**Published:** 2010-03-04

**Authors:** Darren A. Yuen, Kim A. Connelly, Andrew Advani, Christine Liao, Michael A. Kuliszewski, Judy Trogadis, Kerri Thai, Suzanne L. Advani, Yuan Zhang, Darren J. Kelly, Howard Leong-Poi, Armand Keating, Philip A. Marsden, Duncan J. Stewart, Richard E. Gilbert

**Affiliations:** 1 Keenan Research Centre of the Li Ka Shing Knowledge Institute, St. Michael's Hospital, Department of Medicine, University of Toronto, Toronto, Ontario, Canada; 2 Department of Medicine, St. Vincent's Hospital, University of Melbourne, Melbourne, Victoria, Australia; 3 Department of Medicine, Princess Margaret Hospital, University of Toronto, Toronto, Ontario, Canada; 4 Ottawa Health Research Institute, University of Ottawa, Ottawa, Ontario, Canada; University of Cincinnati, United States of America

## Abstract

**Background:**

Most forms of chronic kidney disease are characterized by progressive renal and cardiac fibrosis leading to dysfunction. Preliminary evidence suggests that various bone marrow-derived cell populations have antifibrotic effects. In exploring the therapeutic potential of bone marrow derived cells in chronic cardio-renal disease, we examined the anti-fibrotic effects of bone marrow-derived culture modified cells (CMCs) and stromal cells (SCs).

**Methodology/Principal Findings:**

*In vitro*, CMC-conditioned medium, but not SC-conditioned medium, inhibited fibroblast collagen production and cell signalling in response to transforming growth factor-ß. The antifibrotic effects of CMCs and SCs were then evaluated in the 5/6 nephrectomy model of chronic cardio-renal disease. While intravascular infusion of 10^6^ SCs had no effect, 10^6^ CMCs reduced renal fibrosis compared to saline in the glomeruli (glomerulosclerosis index: 0.8±0.1 *v* 1.9±0.2 arbitrary units) and the tubulointersitium (% area type IV collagen: 1.2±0.3 *v* 8.4±2.0, p<0.05 for both). Similarly, 10^6^ CMCs reduced cardiac fibrosis compared to saline (% area stained with picrosirius red: 3.2±0.3 *v* 5.1±0.4, p<0.05), whereas 10^6^ SCs had no effect. Structural changes induced by CMC therapy were accompanied by improved function, as reflected by reductions in plasma creatinine (58±3 *v* 81±11 µmol/L), urinary protein excretion (9×/÷1 *v* 64×/÷1 mg/day), and diastolic cardiac stiffness (left ventricular end-diastolic pressure-volume relationship: 0.030±0.003 *v* 0.058±0.011 mm Hg/µL, p<0.05 for all). Despite substantial improvements in structure and function, only rare CMCs were present in the kidney and heart, whereas abundant CMCs were detected in the liver and spleen.

**Conclusions/Significance:**

Together, these findings provide the first evidence suggesting that CMCs, but not SCs, exert a protective action in cardio-renal disease and that these effects may be mediated by the secretion of diffusible anti-fibrotic factor(s).

## Introduction

Repair by connective tissue formation is a fundamental response to acute injury. If unchecked, however, the resulting fibrotic response leads to parenchymal replacement and organ dysfunction, estimated to account for nearly 45% of all deaths in the industrialized world [1]. Chronic kidney disease (CKD), for instance, now estimated to affect almost 20 million adults in the United States alone, is characterized by progressive renal fibrosis with attendant reduction in glomerular filtration that ultimately results in the need for dialysis or transplantation to preserve life [2]. Moreover, in CKD, the fibrotic pathology is not confined to the kidney but is also present in the heart [3], where even mild degrees of renal impairment are associated with ventricular stiffening, impaired relaxation and diastolic dysfunction that increase as kidney function worsens [4]. Importantly, diastolic dysfunction is a primary contributor to cardiovascular morbidity and mortality in CKD patients, where its presence portends a particularly poor prognosis [5].

Studies conducted over almost two decades have consistently implicated transforming growth factor-ß (TGF-ß) as a key mediator of pathological fibrosis [6]. Moreover, in addition to its profibrotic effects, TGF-ß has also been implicated in microvascular loss [7], cardiomyocyte hypertrophy [8], and podocyte dysfunction [9] that characterize cardio-renal disease. Indeed, inhibition of TGF-ß has been a major target for drug discovery with several small molecules, antibodies and nucleic acid-based strategies in development [10].

While most experimental and clinical studies of bone marrow derived cell (BMDC) therapy have been undertaken for the treatment of large vessel angio-occlusive disease [11], [12], [13], others have demonstrated that various BMDC populations may exert beneficial effects in other settings also, including fibrosis in the liver [14], an organ in which a dual blood supply renders it ischemia-resistant. With this in mind, we speculated as to whether this effect might be common to the two types of BMDCs that are currently under investigation as potential therapeutic agents: bone marrow-derived culture-modified cells (CMCs) and stromal cells (SCs).

Here we show that CMCs reduce collagen formation, inhibit TGF-ß signaling and also effectively reduce renal and cardiac fibrosis in a rodent model of chronic cardio-renal disease that mimics human disease. Importantly, these effects were associated with improvements in kidney and heart function, an effect not seen with current clinically available therapies.

## Results

### Conditioned Medium from CMCs Inhibits Fibroblast Collagen Production

**Figure 1:**
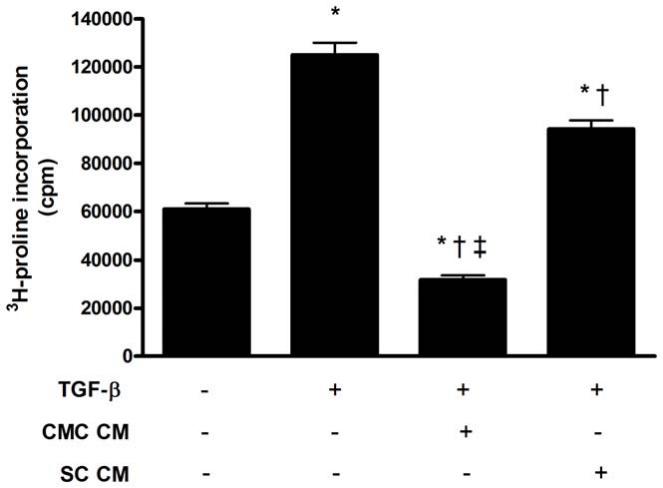
*In vitro* conditioned medium ^3^H-proline incorporation assays (n = 3 independent experiments). * p<0.05 vs. control (no TGF-β, serum-free EBM-2 medium). † p<0.05 vs. serum-free EBM-2 medium + TGF-β. ‡ p<0.05 vs. SC-CM + TGF-β. Abbreviations: CMC CM: CMC conditioned medium. SC CM: SC conditioned medium. TGF-β: transforming growth factor-β. cpm  =  counts per minute.

Progenitor cells have been shown to secrete factors with pro-angiogenic activity that might contribute to their beneficial effects. We therefore considered whether such cells might also secrete factor(s) with anti-fibrotic activity. Accordingly, we incubated CMCs and marrow stromal cells (SCs) in serum-free medium for 24 hours. The effects of these conditioned media on TGF-ß induced collagen production were then compared with serum-free medium (SFM) in a fibroblast assay system. While conditioned medium from SCs had minimal effect, CMC-CM dramatically reduced TGF-β-induced fibroblast ^3^H-proline incorporation, a robust marker of collagen production (Fig. 1).

### Conditioned Medium from CMCs but Not SCs Inhibits TGF-β Signalling

**Figure 2:**
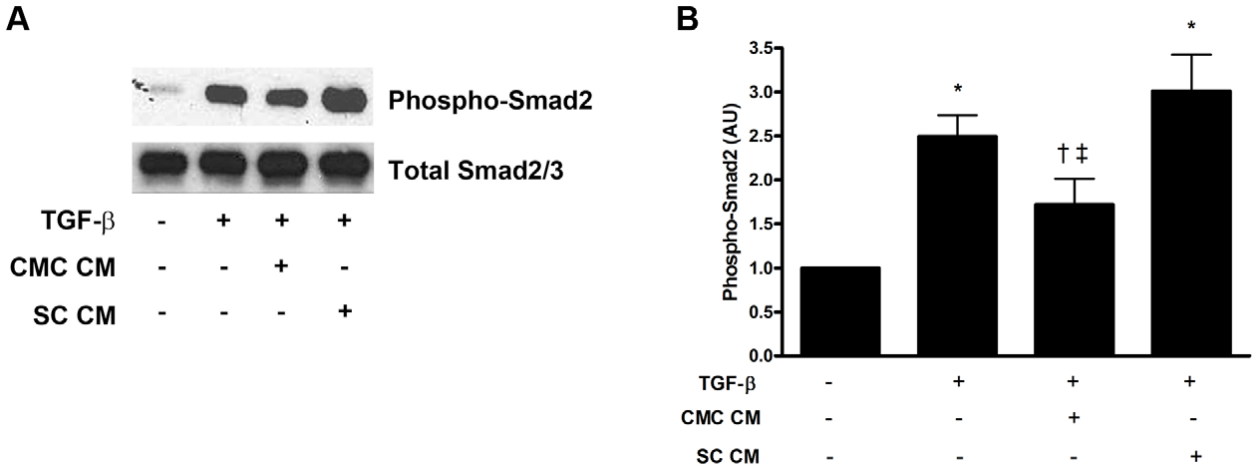
Intracellular TGF-β-induced Smad2 signalling. (a) Representative Western blot for phospho-Smad2 and total Smad2/3. (b) Quantitative analysis of Western blot for phospho-Smad2 (n = 5 independent experiments). * p<0.05 vs. control (no TGF-β, serum-free EBM-2 medium). † p<0.05 vs. TGF-β + serum-free EBM-2 medium. ‡ p<0.05 vs. SC-CM + TGF-β. Abbreviations: CMC CM: CMC conditioned medium. SC CM: SC conditioned medium. TGF-β: transforming growth factor-β. AU: arbitrary units.

To further explore the effects of the conditioned media on TGF-ß induced collagen production, we considered whether medium derived from CMCs might inhibit the early stages of the TGF-ß signalling cascade by examining phosphorylation of Smad2. As expected, TGF-β stimulated robust phosphorylation of intracellular Smad2 in cultured fibroblasts. While conditioned medium from SCs had minimal effect, CMC-CM significantly reduced TGF-β-induced Smad2 phosphorylation (Fig. 2a–b).

### CMC Infusion Attenuates Renal Dysfunction in the SNX Rat

Given the potent antifibrotic effects of CMCs *in vitro*, we next examined the effects of CMC infusion *in vivo* using the 5/6 subtotally nephrectomized (SNX) rat as a model of progressive, non-immune-mediated chronic kidney disease that also develops heart failure. In this model, the kidney and heart develop progressive fibrosis similar to many patients with CKD. Systolic blood pressure (SBP), urinary protein excretion and plasma creatinine were all elevated in SNX when compared with sham-operated animals at 8 weeks (Table 1). The kidneys receive approximately 20% of cardiac output such that infusing cells intra-arterially would be expected to deliver a greater number of cells to this site of injury than might be anticipated from intravenous administration. We therefore compared intra-arterial with intravenous routes of cell administration, with an *a priori* decision that if similar reductions in proteinuria were apparent, then the two groups would be considered as one, enabling greater statistical power for comparison with control animals. Administration of 10^6^ CMCs attenuated the magnitude and progression of proteinuria in SNX rats to a near-identical extent whether infused by an intravenous or intra-arterial route. Indeed, for a range of functional and structural parameters in both the kidney and heart, outcomes were nearly identical for both routes of administration (Fig. S1). In addition to reducing proteinuria, cell therapy attenuated the increase in plasma creatinine in SNX rats, though it remained elevated in comparison to sham animals (Table 1). No mortality was seen in any treatment group after CMC or saline infusion.

**Table 1:** Renal function at 8 weeks post-surgery.

	Sham	SNX	SNX–CMC iv	SNX–CMC ia	SNX - CMC
*N*	6	15	9	8	17
Urine protein (mg/day)	3.1×/÷1.1	64.0×/÷1.4*	9.3×/÷1.4†	8.8×/÷1.7†	9.0×/÷1.3†
Plasma creatinine (µmol/L)	32±2	81±11*	55±3†	62±5*	58±3* †
Systolic BP (mm Hg)	123±2	184±10*	172±6*	178±6*	175±4*

Abbreviations: SNX, SNX animal treated with phosphate-buffered saline; SNX–iv, SNX animal treated with intravenous CMC infusion; SNX–ia, SNX animal treated with intra-arterial CMC infusion; SNX–CMC, SNX animal treated with either intravenous or intra-arterial CMC infusion. Urine protein excretion is presented as geometric mean ×/÷ tolerance factors.

*p<0.05 versus sham-operated animals.

†p<0.05 versus SNX animals.

### CMC Infusion Ameliorates Fibrosis and Microvascular Rarefaction in the SNX Rat

**Figure 3:**
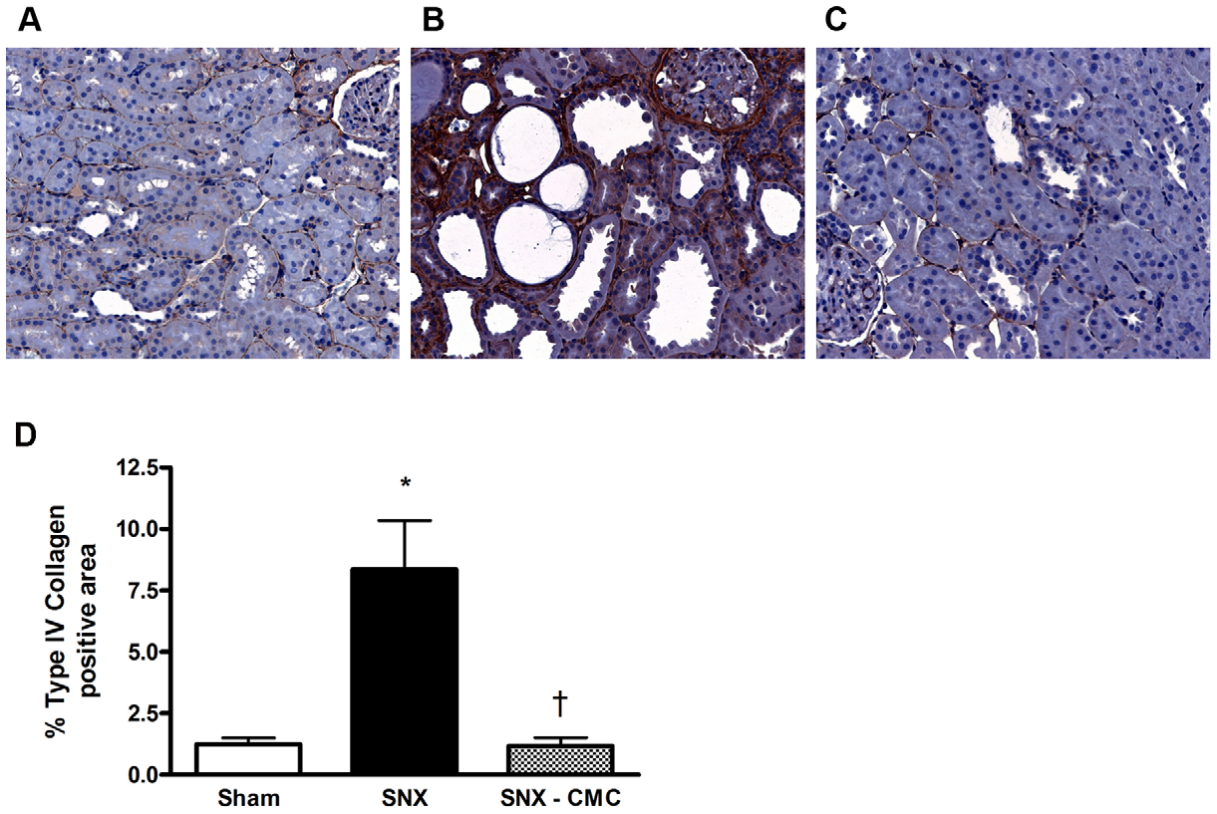
Tubulointerstitial renal cortical fibrosis. Kidney sections were immunolabelled for Type IV collagen 8 weeks post-surgery. (a–c) Representative cortical tubulointerstitial images. Original magnification × 160. (a) Sham animal. (b) SNX animal. (c) SNX–CMC animal. (d) Type IV collagen % positivity. * p<0.05 vs. sham operated animals. † p<0.05 vs. SNX animals.

**Figure 4:**
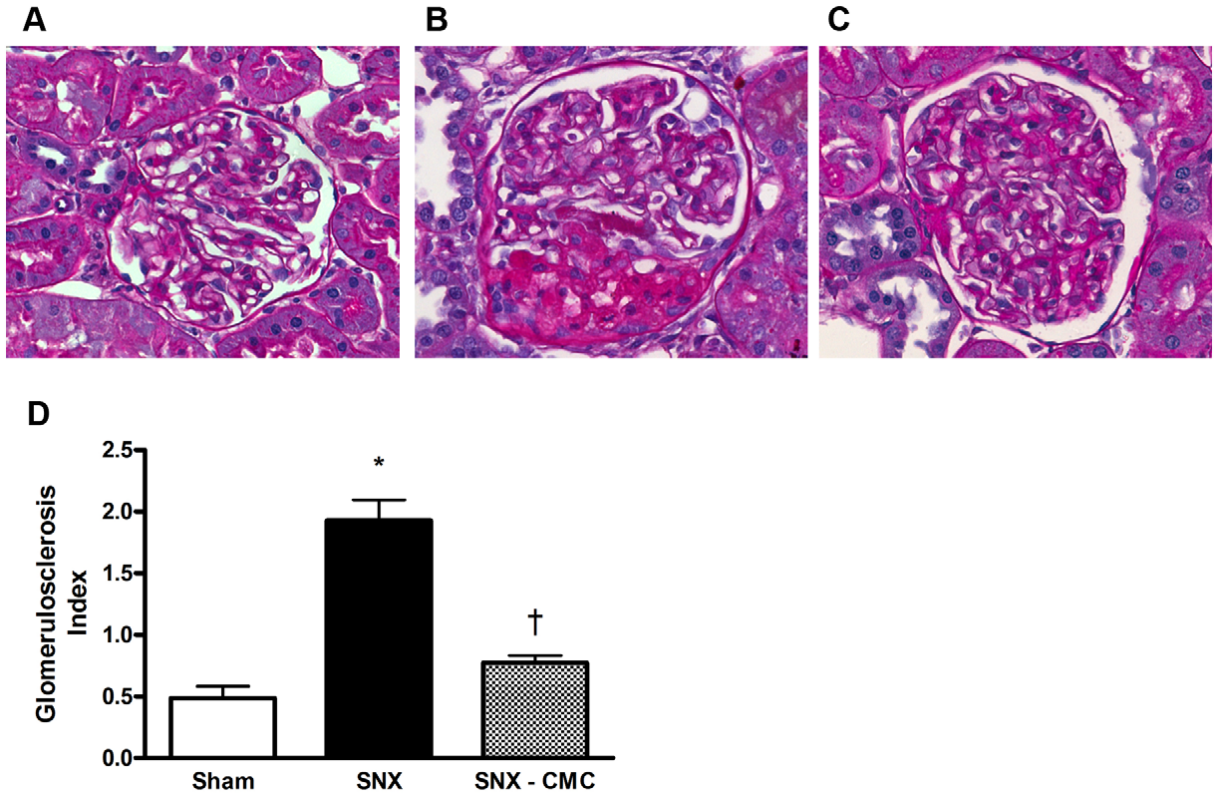
Glomerulosclerosis. PAS-stained kidney sections at 8 weeks post-surgery were used to assess the degree of glomerulosclerosis. (a–c) Representative glomerular images. Original magnification × 400. (a) Sham animal. (b) SNX animal. (c) SNX–CMC animal. (d) Glomerulosclerosis index (GSI). * p<0.05 vs. sham operated animals. † p<0.05 vs. SNX animals.

To determine the structural correlates of the improved kidney function seen with CMC therapy, we examined the extent of glomerulosclerosis and tubulointerstitial fibrosis in SNX animals treated with syngeneic CMCs. Tubulointerstitial fibrosis, a major correlate of declining kidney function, was substantially increased in SNX rats, as was glomerulosclerosis. CMC-treated rats showed substantial reductions in the extent of fibrosis, both in the tubulointerstitium (Fig. 3) and in the glomerulus (Fig. 4).

**Figure 5:**
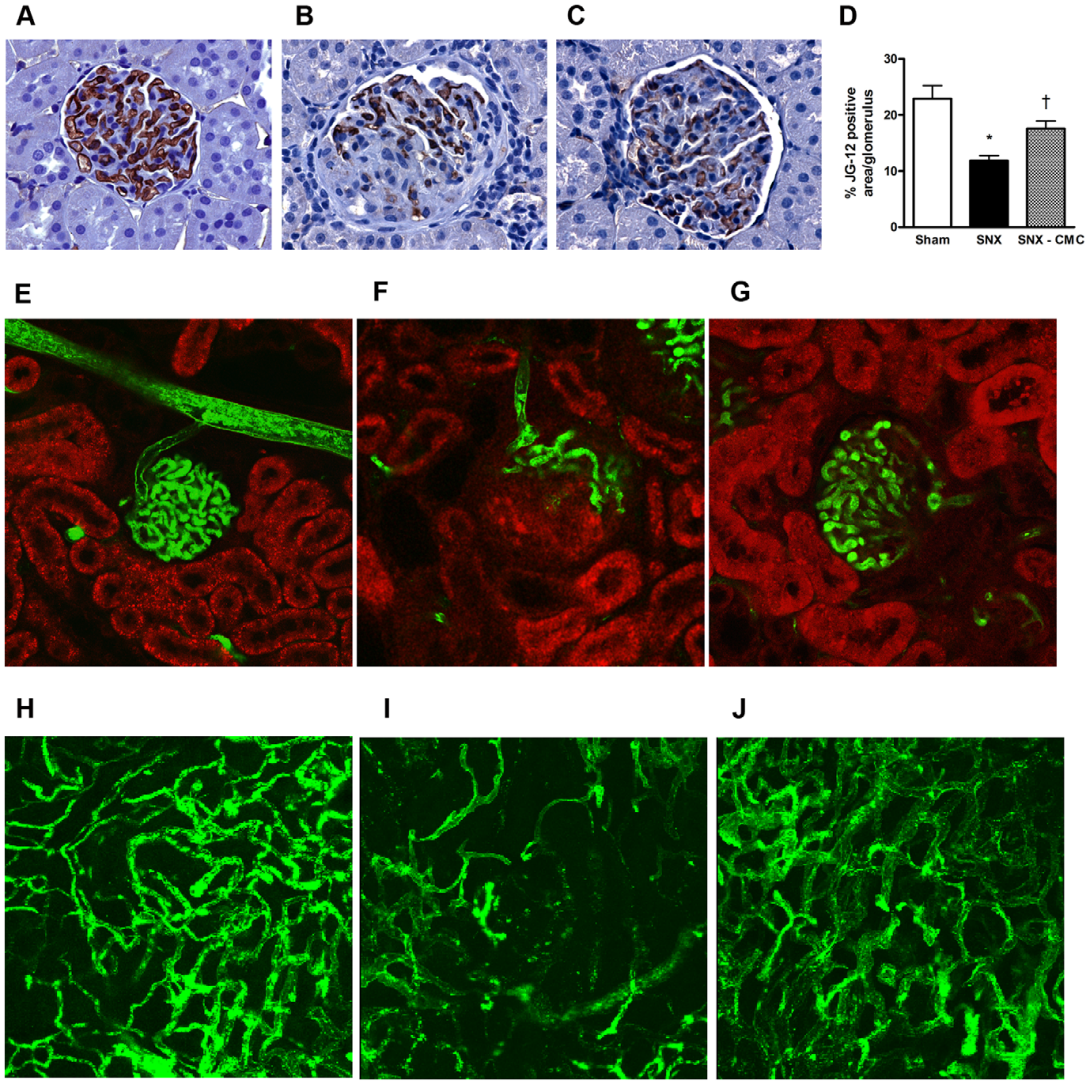
Renal microvasculature. (a–c) Kidney sections were immunolabelled with JG-12 antibody, which recognizes a glomerular endothelial cell antigen. Representative JG-12 immunostained images. Original magnification × 400. (a) Sham animal. (b) SNX animal. (c) SNX–CMC animal. (d) % positive area for JG-12 immunostaining per glomerulus. (e–g) Representative fluorescence microangiography (FMA) images of glomeruli. The autofluorescence of the surrounding renal cortex was captured and pseudocolourized red. The green fluorescence of the infused beads outlines microvascular structures. Original magnification × 40. (e) Sham animal. (f) SNX animal. (g) SNX–CMC animal. (h–j) Representative FMA images of peritubular capillaries. Each image is a flattened Z-stack of a 100 µm section taken with a confocal microscope. Original magnification × 75. (h) Sham animal. (i) SNX animal. (j) SNX–CMC animal. * p<0.05 vs. sham operated animals. † p<0.05 vs. SNX animals.

Progressive fibrosis replaces normal renal parenchyma, including its constituent capillaries. Accordingly, we examined glomerular capillary density using immunolabelling for a glomerular endothelial cell antigen in SNX and CMC-treated SNX-rats. As expected, SNX rats experienced a significant reduction in glomerular capillary density, while CMC-treated SNX rats demonstrated preservation of glomerular capillaries (Fig. 5). With the consideration that the increased abundance of cells showing positive endothelial cell immunolabelling in CMC-treated SNX rats may reflect augmentation in antigenic expression rather than an increase in capillarity, a subset of SNX and CMC-treated SNX animals underwent fluorescence microangiography (FMA), demonstrating loss of both glomerular and peritubular capillaries (Fig. 5), along with their amelioration by CMC administration.

### Chronic Diastolic Cardiac Dysfunction Is Improved by CMC Administration

**Figure 6:**
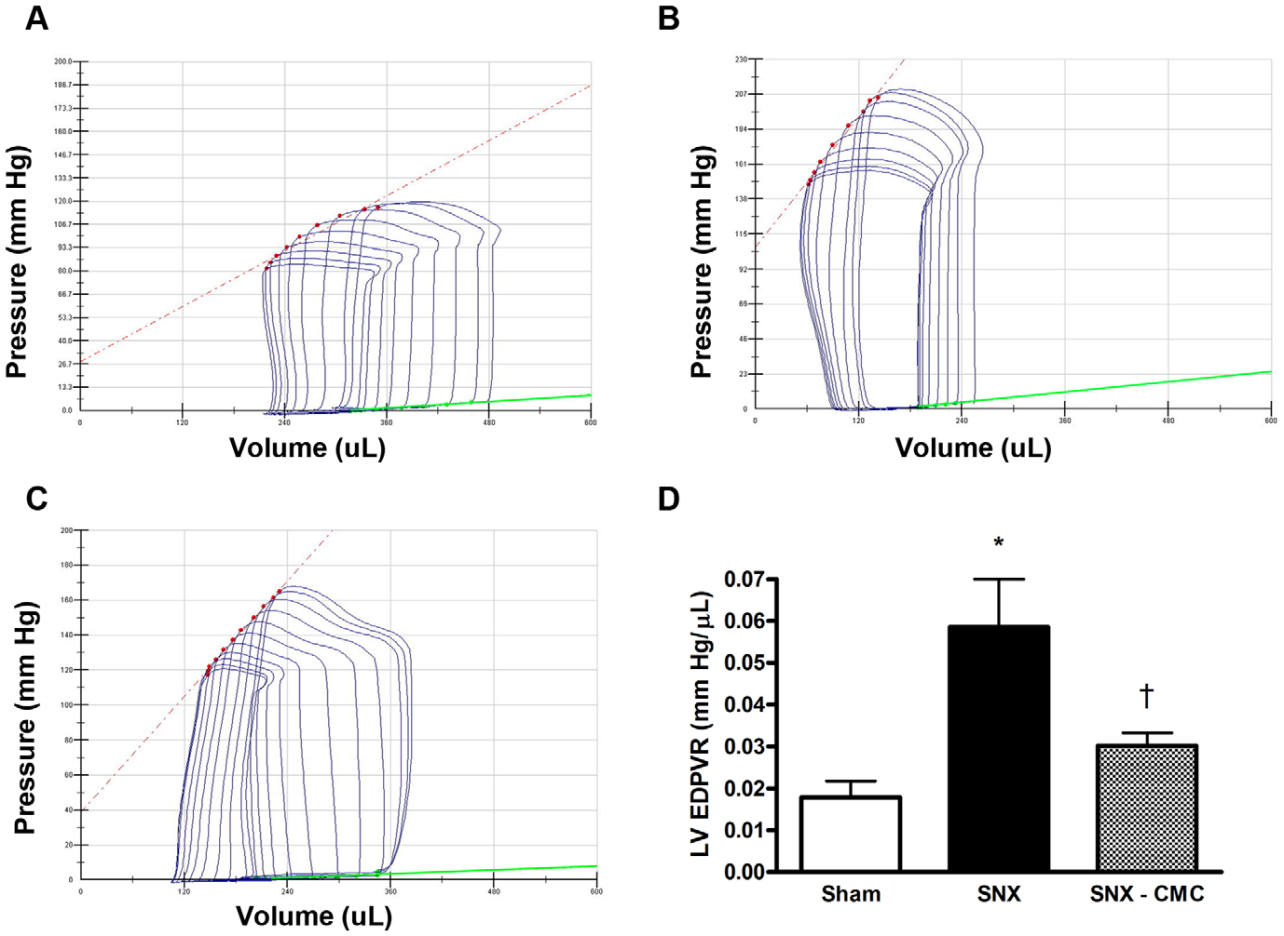
Invasive cardiac hemodynamic assessment at 8 weeks post-surgery. (a–c) Representative pressure-volume (PV) loops obtained 8 weeks post-surgery. Each loop is generated by the simultaneous measurement of left ventricular pressure and volume at all points in the cardiac cycle, enabling the measurement of cardiac functional parameters. Left ventricular end diastolic pressure volume relationship (LV EDPVR), a measure of passive LV relaxation, is represented by the slope of the tangent of the base of each PV loop (green lines). (a) Sham animal. (b) SNX animal. (c) SNX–CMC animal. (d) Quantitative analysis. * p<0.05 vs. sham operated animals. † p<0.05 vs. SNX animals.

Given the close association between kidney disease and heart failure, and the importance of chronic heart failure as a poor prognostic factor in CKD patients, we next examined pre-terminal (8 weeks post-surgery) cardiac function in SNX rats with and without CMC therapy, focussing particularly on diastolic function. SNX rats demonstrated chronic impairment of diastolic function as demonstrated by an elevated left ventricular end-diastolic pressure volume relationship (LV EDPVR), a marker of impaired left ventricular compliance (Fig. 6). When compared with untreated SNX animals, CMC-treated animals showed improvement in passive diastolic filling, as manifested by a reduction in LV EDPVR (Fig. 6).

### CMC Infusion Ameliorates Cardiac Structural Damage in the Remnant Kidney Rat

**Figure 7:**
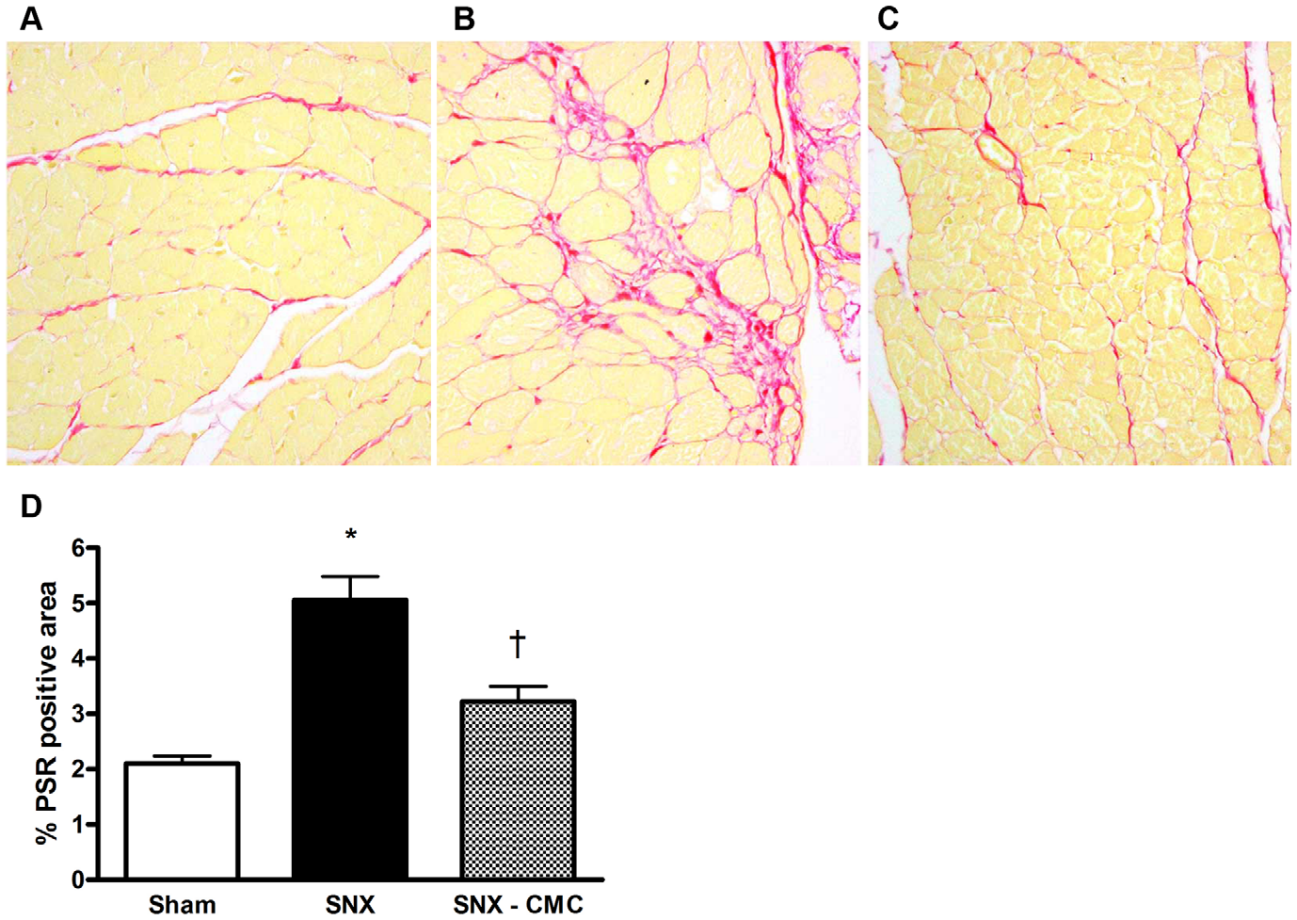
Cardiac fibrosis. (a–c) Representative picrosirius red stained heart sections at 8 weeks post-surgery. Original magnification × 160. (a) Sham animal. (b) SNX animal. (c) SNX–CMC animal. (d) Quantitative cardiac fibrosis analysis. * p<0.05 vs. sham operated animals. † p<0.05 vs. SNX animals. Abbreviations: PSR: picrosirius red.

**Figure 8:**
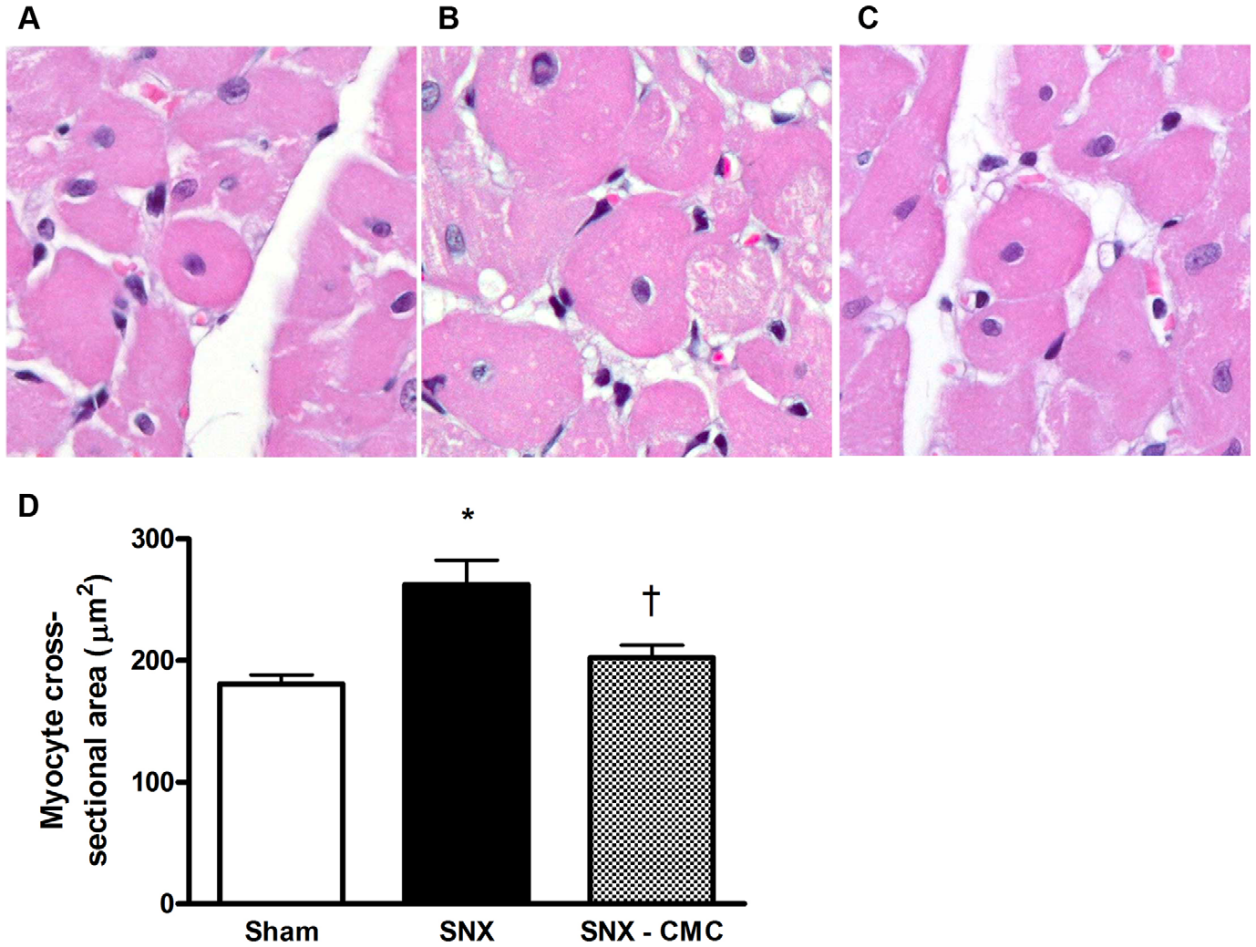
Cardiomyocyte cross-sectional area 8 weeks post-surgery. Original magnification × 400. (a) Sham animal. (b) SNX animal. (c) SNX–CMC animal. (d) Quantitative analysis of myocyte cross-sectional area. * p<0.05 vs. sham operated animals. † p<0.05 vs. SNX animals.

One of the primary determinants of impaired left ventricular relaxation is the development of cardiac fibrosis. Accordingly, we examined the degree of cardiac matrix accumulation at 8 weeks post-surgery. As in the kidney, SNX lead to significant interstitial fibrosis in the heart. CMC treatment significantly attenuated the extent of cardiac fibrosis (Fig. 7). In addition, myocyte cross sectional area, indicative of cellular hypertrophy and thus worsened cardiac afterload, was increased in SNX rats and reduced by CMC administration (Fig. 8).

### SCs Were without Effect on Cardio-Renal Function and Structure in the Remnant Kidney Rat

To ascertain whether the beneficial actions of CMCs in the SNX model tracked with their *in vitro* anti-fibrotic effects or whether it reflected a more generalizable attribute of bone marrow derived cells, we next examined the *in vivo* efficacy of marrow stromal cells in the SNX model. In contrast to CMCs, intravascular infusion of 10^6^ SCs did not attenuate the structural or functional abnormalities in either the heart or kidney of SNX rats (Table S1 and Fig. S2).

### *In Vivo* Tissue Localization of Infused CMCs

**Figure 9:**
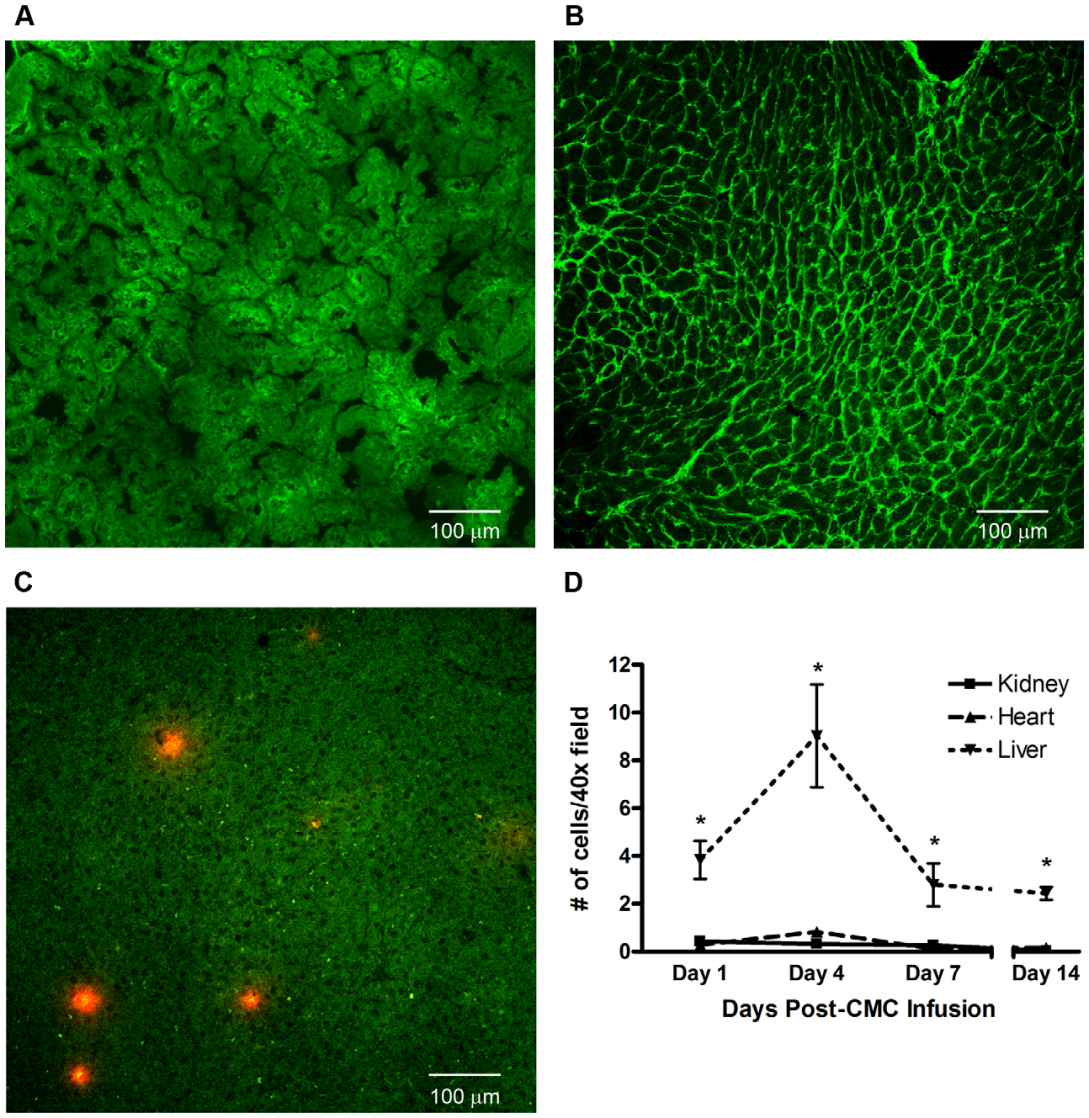
*In vivo* CMC tissue distribution 4 days post-infusion. (a–c): Representative confocal microscopy images of kidney, heart, and liver respectively at 4 days post-CMC infusion. Original magnification × 20. (a) Kidney cortex. (b) Heart. (c) Liver. (d) Time course of CMC retention in kidney, heart, and liver. n = 3 animals per time point. * p<0.05 vs. kidney.

Given that both intravenous and intra-arterial CMC delivery had equivalent effects, and our *in vitro* results suggesting that CMCs release soluble antifibrotic factor(s) that inhibit TGF-β signalling in fibroblasts, we next sought to examine the abundance of the administered cells in the kidney and heart. Accordingly, CMCs were loaded with the vital cytoplasmic fluorophore CMTMR, and infused by tail vein injection into rats 4 weeks after 5/6 subtotal nephrectomy. While only rare CMTMR-labelled CMCs were detected in the kidneys and hearts of infused SNX animals at either early (1 day) or later (14 day) time points, CMTMR-labelled CMCs were abundant in the liver and spleen with a notable peak 4 days after administration but persistence throughout the time course of the study (Fig. 9).

## Discussion

Despite current treatment, the incidence of end-stage renal disease continues to rise, with no major therapeutic advances to stall the progression of CKD since the introduction of renin-angiotensin system antagonists. In the present study, we show that declining GFR and worsening proteinuria, two of the cardinal laboratory indicators of progression towards end-stage kidney disease, were substantially attenuated by the administration of culture modified bone marrow derived cells in a widely used model of non-immune chronic kidney disease. Moreover, chronic heart failure, a common comorbidity in CKD that portends a poor prognosis, was also improved by cell therapy.

While CMCs have therapeutic effects in a range of diseases, the mechanisms by which they do so remain unclear. In the present study we explored their anti-fibrotic activity, focusing on the dominant prosclerotic factor TGF-ß, and the cells' *in vivo* efficacy in a model of chronic kidney disease that is characterized by clinically significant progressive scarring of both the kidney and the heart. CMCs, but not SCs, were shown to secrete factor(s) that inhibit TGF-ß receptor signalling and collagen production in cultured cells. Intriguingly, the beneficial effects seen *in vivo* with CMC infusion occurred despite minimal CMC localization to the kidney or heart, in either the acute or later settings, suggesting that diffusible factor(s), such as those identified in culture, may also be active in the *in vivo* setting. Indeed, rather than homing to the site of injury, exogenous CMCs were present in large numbers in the liver and spleen, suggesting that they may be preferentially sequestered by the reticulo-endothelial system.

In line with our limited understanding of how non-embryonic cell therapy might work, there has been considerable conjecture as to whether the administered cells differentiate [15], fuse with resident cells [16] or secrete paracrine factors [17]. Notwithstanding this debate, their efficacy has nevertheless been assumed to be dependent on cell homing to sites of injury [18]. As a consequence, techniques for increasing local cell delivery have been used increasingly in clinical trials in an attempt to maximize the therapeutic gain [19]. However, two studies in acute kidney injury models have suggested that circulating factors elaborated by bone marrow derived cells may account for the beneficial effects observed [20], [21]. In the present study, we compared intravenous with intra-arterial CMC delivery, noting similar efficacy in a range of outcome measures with either mode of administration. Moreover, despite the observed benefits, CMCs were rarely present in the kidneys or hearts of treated SNX animals, whereas high numbers were found in the liver and spleen, suggesting that at least some of their effects may be mediated at a distance, possibly through the elaboration of diffusible factor(s) that inhibit(s) the actions of TGF-ß.

Glomerulonephritis, diabetes and hypertension account for the majority of chronic kidney disease in the developed world. However, despite this diverse range of etiologies, they are often characterized histopathologically by chronic cardiorenal fibrosis and microvascular rarefaction. Indeed, it is the extent of the fibrosis, particularly in the renal interstitium, that correlates most closely with the decline in kidney function [22]. Similarly, in the heart, interstitial fibrosis, along with myocyte hypertrophy and capillary loss, are key components of adverse cardiac remodelling that affect cardiac geometry and function, particularly during diastole [23]. As in the kidney, fibrosis appears as a final common pathway in most forms of chronic cardiac disease, being present in diabetic [24] and uremic cardiomyopathies [25], ischaemic heart disease [23], and even in valvular heart disease where the extent of fibrosis correlates closely with left ventricular end-diastolic pressure and inversely with ejection fraction [26].

Consistent with its spectrum of activity in stimulating fibrosis, hypertrophy, podocyte injury, and capillary loss, elevated TGF-ß expression has been repeatedly demonstrated in studies of cardiac and renal disease in both humans and animal models [6], and in particular the 5/6 subtotally nephrectomised rat [27]. The central role of TGF-β in promoting fibrovascular pathology and subsequent organ dysfunction has been reinforced by multiple studies demonstrating the beneficial effects of blocking TGF-β for either chronic renal [28], [29] or cardiac disease [30], [31]. In the present study, we used ^3^H-proline incorporation as a bioassay of fibroblast collagen production. In these studies, medium derived from CMCs, but not that from SCs, was found to suppress TGF-ß induced ^3^H-proline incorporation, suggesting that soluble factor(s) released by CMCs, at least in part, mediate an anti-fibrotic effect.

To investigate how the culture modified medium was inhibiting TGF-ß induced ^3^H-proline incorporation, we examined its well-described signalling pathway. Binding of TGF-ß to its type 2 receptor (TßR2), permits recruitment and activation of the type 1 receptor (TßR1), which in turn continues the signalling relay by phosphorylating and thereby activating the intermediate Smad2 [32]. In the present study, we show that conditioned medium from cultured CMCs reduced TGF-β-induced Smad2 phosphorylation, suggesting that factor(s) secreted into the medium by CMCs inhibited the activity of the TGF-ß receptor complex. In contrast to CMCs, conditioned medium from cultured SCs had comparatively minimal effects on TGF-ß induced ^3^H-proline incorporation and Smad2 phosphorylation, suggesting that CMCs, and not SCs, are one of the principal BMDC populations that secrete these inhibitory factor(s).

A wide range of methods have been used to isolate and identify progenitor cells in bone marrow [33]. In the absence of specific and unique cell surface markers, we compared two major types of bone marrow derived cells based primarily on their *in vitro* adhesion and growth characteristics. Bone marrow cells were cultured on fibronectin-coated plates for 7 to 10 days, at which time they displayed many of the phenotypic and surface marker characteristics that are commonly associated with endothelial progenitor cells [33]. However, given the ambiguity surrounding the nomenclature of such cells, we used a more operational definition, referring to the cells that we used as early outgrowth, bone marrow derived, culture-modified cells (CMCs). The other broad category of bone marrow derived cells under investigation, mesenchymal or marrow stromal cells (SCs) were identified in this study by their plastic adherence and their ability to differentiate into osteoblasts, adipocytes, and chondroblasts, criteria used widely for their definition [34]. Since both the CMC and SC populations used in our studies were heterogeneous, the generalizability of our findings to other cell doses and other types of bone marrow derived cells, cultured under different conditions or administered in other disease settings, is uncertain.

Clinically, perhaps the most well known link between TGF-ß, fibrosis and renal dysfunction relates to angiotensin II, as agents that block its formation and receptor binding are the cornerstones of current CKD management. While angiotensin converting enzyme (ACE) inhibitors and angiotensin receptor blockers (ARBs) were initially viewed as exerting their beneficial effects through primarily hemodynamic means, such as their ability to reduce intraglomerular pressure, more recent studies have emphasized the ability of these agents to reduce TGF-ß formation and attenuate fibrosis in the tubulointerstitium as well as the glomerulus, in both humans [35], [36] and experimental animals [27], [37]. Similarly, in the heart, beyond its haemodynamic effects, the ability of angiotensin II to induce TGF-ß expression [38] is thought to be a major contributor to the beneficial effects of renin-angiotensin system (RAS) blockade in heart failure [39], [40].

To date, many of the studies showing beneficial effects of bone marrow derived cells have focussed on their pro-angiogenic actions. Consistent with these reports, the present study also showed that cell therapy improved the microvasculature of the kidney as assessed by both immunohistochemical and angiographic techniques. While loss of the capillary microvasculature is a feature of the SNX model, as with CKD in humans, growth factors with primarily angiogenic effects, such as vascular endothelial growth factor (VEGF), do not attenuate proteinuria, hypertension or glomerulosclerosis despite preventing microvascular loss [41]. In contrast to VEGF, agents that target the pro-sclerotic factor TGF-β, either directly [29] or indirectly [28], have been shown to attenuate not only fibrosis and declining kidney function, but to also reduce proteinuria in models of CKD. Similarly, in the heart, agents that inhibit TGF-ß activity have also been shown to reduce fibrosis and cardiac dysfunction [30], [31], [42].

Functioning in tandem, the kidney and heart provide the physiological regulation of extracellular fluid volume, sodium homeostasis, blood pressure, cardiac output and glomerular filtration rate (GFR). In the setting of kidney disease, the resulting dysregulation affects cardiac function with increases in both pre- and afterload, along with the accumulation of waste products that characterizes the uraemic state. At a histopathological level, fibrosis and myocyte hypertrophy are features of cardiac tissue from both humans and animals with impaired kidney function that are thought to mediate the abnormal relaxation properties of the left ventricle in CKD [25], [43], [44]. In the present study, we found that CMC administration not only attenuated these pathological changes in cardiac structure but also led to improvements in diastolic filling. Importantly, these changes in cardiac function were assessed using load-insensitive techniques, an important consideration in the setting of the alterations in blood volume and arterial pressure seen in CKD.

As commonly seen with other therapeutic interventions in both the experimental and clinical settings, CMC administration led to a more dramatic reduction in proteinuria than in serum creatinine, reflecting the different pathogeneses of these two disease manifestations as well as the non-linear relationship between serum creatinine and GFR. Interestingly, while improvements in kidney filtration and proteinuria were observed with CMC treatment, systemic arterial pressure was unaffected, remaining significantly elevated above that of sham animals, as has been documented with other anti-TGF-β therapies in experimental renal disease [28]. These data suggest that the mechanisms underlying CMC-induced renal structural and functional changes in the 5/6 nephrectomy model are mostly independent of changes in systemic blood pressure. However, a modest trend towards blood pressure reduction was seen in CMC-treated animals at the end of the study, suggesting that this was a consequence of improved glomerular filtration rather than a cause. Importantly, given the well-recognized importance of systemic blood pressure control in the attenuation of renal disease progression [45], our data suggests that CMC infusion may provide additive benefit when used in combination with antihypertensive therapy.

In summary, to our knowledge, this study is the first to document the beneficial effects of exogenous CMCs in reducing the functional and structural manifestations of both renal and cardiac injury in a well-established model of chronic progressive cardio-renal disease. This study also suggests that CMCs may exert therapeutic effects not solely through localization to injured organs, but also from remote locations, and that specifically their anti-fibrotic activity may be mediated by the elaboration of soluble factor(s) that inhibit TGF-ß signalling.

## Materials and Methods

### Ethics Statement

All animal work was conducted according to the Canadian Council on Animal Care guidelines [46]. The specific experimental protocols were approved by the Animal Care Committee of St. Michael's Hospital.

### CMCs and SCs Were Cultured from Bone Marrow Cells of Syngeneic Male F344 Rat Donors

CMCs were cultured as previously described [47]. Briefly, bone marrow cells were flushed from the femora and tibiae of 4 week old male Fischer 344 rats with sterile phosphate-buffered saline (PBS). The collected cells were centrifuged at 360 rcf for 10 min at 22°C. The supernatant was discarded, and the cell pellet was resuspended in differential endothelial cell culture medium (EGM-2, Clonetics Corp, Walkersville, MD). The cells were plated on human fibronectin-coated tissue culture flasks and incubated at 37°C with 5% CO_2_ for 7 to 10 days to produce CMCs. Medium was changed every 2–3 days. Adherent CMCs adopted a typical cobblestone appearance in culture, and displayed positivity for a range of endothelial cell markers including binding of *Bandeiraea simplicifolia*-1 lectin, uptake of fluorescently labelled acetylated-LDL (DiI acLDL, Molecular Probes) and immunostaining for von Willebrand Factor (vWF) and vascular endothelial growth factor receptor type 2 (VEGFR-2), as previously described [47].

SCs were cultured as previously described [48]. Briefly, flushed bone marrow cells harvested as above were centrifuged at 360 rcf for 10 minutes at 22°C. The supernatant was discarded, and the cell pellet was resuspended in PBS supplemented with 0.5% bovine serum albumin and 2 mM EDTA (pH 7.2). The cells were then resuspended in Dulbecco's Modified Eagle Medium (DMEM, Gibco™; Invitrogen, Grand Island, NY), plated on plastic tissue culture flasks, and incubated at 37°C with 5% CO_2_ to produce mesenchymal stromal cells (SCs). Medium was changed every 2–3 days. Cells used for experiments were between passages 2 and 6. The phenotype of the cells was confirmed through their plastic adherence and their ability to differentiate into chondroblasts, osteoblasts, and adipocytes [34].

### Conditioned Medium Generation

CMC conditioned medium was generated by incubating subconfluent 10 day cultured CMCs with serum-free endothelial basal medium-2 (EBM-2) medium for 24 hr as previously described [17]. The medium was then collected and used for *in vitro* experiments. SC conditioned medium was generated similarly by incubating subconfluent passage 2–6 SCs with DMEM. Serum-free EBM-2 medium served as a control.

### ^3^H-Proline Incorporation Assay

Neonatal cardiac fibroblasts were isolated from the hearts of 1-day-old Sprague-Dawley rat pups as previously described [42]. Cells were passaged twice and then seeded at a density of 50,000/well in DMEM. After 24 hr, fibroblasts were serum starved in DMEM supplemented with 0.5% bovine serum albumin (BSA), 150 uM L-ascorbic acid (Sigma-Aldrich), and 1% antibiotic/antimycotic mixture (Gibco™, Invitrogen), and then incubated with 0.5 mL of conditioned or unconditioned medium for 4 hr. For TGF-β stimulation experiments, TGF-β was added at a concentration of 20 ng/mL after the 4 hr pre-incubation with conditioned or unconditioned medium. The fibroblasts were then incubated with [^3^H]-proline (1 µCi/well, L-[2,3,4,5-^3^H]-proline; Amersham Biosciences) for 44 hours. Fibroblasts were then harvested, washed four times with PBS, solubilised in 0.75 ml of 1 M NaOH and then neutralized with 0.5 ml 1 M HCl. Incorporation of exogenous [^3^H]-proline was measured using a liquid scintillation counter (LS 6000 Beckman Instruments, Inc) as previously described [49].

### Western Blot Analysis

Neonatal cardiac fibroblasts were isolated and cultured as described above. After 24 hrs, fibroblasts were serum starved in DMEM supplemented with 0.5% bovine serum albumin (BSA), and 1% antibiotic/antimycotic mixture (Gibco™, Invitrogen), and then incubated with 0.5 mL of unconditioned medium, CMC CM, or SC CM for 4 hours. The fibroblasts were then stimulated with TGF-β 20 ng/mL for 2 hrs, and then lysed with Cell Lysis Buffer (Cell Signaling Technology). Cell lysates were probed for Smad2/3 (rabbit anti-rat, Catalog No. 3102) and phospho-Smad2 (rabbit anti-rat, Catalog No. 3104) using antibodies from Cells Signaling Technology after SDS-PAGE.

### Animals

Recipient (age 8–10 wks) and donor (age 3–4 wks) Fischer 344 rats were obtained (Charles River, Montreal, Quebec). Rats were maintained at the St. Michael's Hospital Animal Research Vivarium in a temperature-controlled (22°C) room with *ad libitum* access to commercial standard rat chow. All animal studies were approved by the St. Michael's Hospital Animal Ethics Committee in accordance with the Guide for the Care and Use of Laboratory Animals (NIH Publication No. 85-23, revised 1996).

### Subtotal Nephrectomy Animal Model

Fischer 344 rats (Charles River, Montreal, Quebec) of 8 weeks of age were randomized to one-step subtotal 5/6 nephrectomy (n = 43) or sham surgery (n = 6), as previously described [27]. Briefly, animals were anesthetized with inhaled 2.5% isoflurane. The right kidney was removed via subcapsular nephrectomy. Infarction of approximately two thirds of the left kidney was achieved via selective ligation of 2 out of the 3 or 4 branches of the renal artery. Sham surgery consisted of laparotomy and manipulation of both kidneys before wound closure.

### Cell Infusion

Four weeks after SNX, animals were randomized in a 1∶1∶2 fashion via a masked protocol to receive an infusion of: (1) 1×10^6^ CMCs (intra-arterial injection), (2) 1×10^6^ CMCs (intravenous injection), or (3) sterile phosphate-buffered saline (PBS, intravenous injection). For this procedure, animals were anesthetized with inhaled 2.5% isoflurane and either the left internal carotid artery (intra-arterial route) or right internal jugular vein (intravenous route) was cannulated with a PE50 catheter with the tip placed at the aortic arch or in the superior vena cava respectively. For PBS controls, PBS was infused via a single tail vein injection. On the day of infusion, CMCs were trypsinized and resuspended at a concentration of 10^6^ cells/mL in sterile PBS. The total volume of infusion for each animal was 1 ml.

The *in vivo* study was predicated on the assumption that a clinically relevant effect of CMCs would be similar to that of angiotensin converting enzyme inhibitor therapy, with which an approximate 50% reduction in proteinuria is seen in the SNX model [27]. Having postulated that the intra-arterial and intravenous routes would result in similar reductions in proteinuria, we planned *a priori* should this be the case, the two intervention groups would be considered as one.

In a second cell infusion study, a further group of animals received 1×10^6^ SCs by intravenous injection, as described above, using passage 2–6 SCs that had been trypsinized and resuspended in 1 mL of sterile PBS prior to administration.

### Renal Functional Parameters

Body weight was determined weekly. Every two weeks, rats were individually housed in metabolic cages for 24 hr for subsequent determination of urine protein excretion using the benzethonium chloride method. Plasma creatinine was determined at sacrifice by the Jaffé method. Systolic blood pressure was measured in conscious rats using an occlusive tail-cuff plethysmograph attached to a pneumatic pulse transducer (Powerlab, ADInstruments, Colorado Springs, CO), as previously described [50].

### Cardiac Catheterization

Cardiac catheterization and data analysis were performed as previously published [51]. In brief, a 2F miniaturized combined conductance catheter-micro-manometer (Model SPR-838 Millar instruments, Houston, TX) was inserted into the carotid artery to obtain aortic blood pressure, then advanced into the left ventricle until stable PV loops were obtained. Data were then acquired under steady state conditions and during preload reduction. The following functional parameters were then calculated (Millar analysis software PVAN 3.4): end diastolic volume, end diastolic pressure, end systolic pressure, ejection fraction, the slope of the end systolic pressure volume relationship, and the slope of the end diastolic pressure volume relationship (EDPVR).

### Tissue Preparation and Histochemistry

At study end, rats were anesthetized with inhaled isoflurane 2.5%. The left renal artery was clamped and the remnant kidney removed, decapsulated, and sliced transversely before immersion fixation in 10% neutral buffered formalin, embedding in cryostat matrix (Tissue-Tek, Sakura, Kobe, Japan), or flash freezing in liquid nitrogen. The heart, liver, and spleen were also excised and stored in a similar manner. Formalin-fixed tissues were routinely processed, embedded in paraffin, and sectioned before staining with haematoxylin & eosin, Periodic acid-Schiff, and/or picrosirius red stains.

### Immunohistochemistry

Glomerular endothelial cells were immunostained using the mouse anti-rat monoclonal antibody JG-12 (Bender Medsystems), which binds to endothelial cells of blood vessels but not to lymphatics in rat kidney [50]. Tubulointerstitial fibrosis was assessed by examining the accumulation of collagen type IV within the renal cortical tubulointerstitium. Type IV collagen deposition was detected by a goat anti-rat type IV collagen polyclonal antibody (Southern Biotech, Birmingham, AL), and quantified in six random non-overlapping 20x fields for each animal in a blinded fashion as previously described [52]. Immunohistochemistry was performed as previously described [53]. Omission of primary antisera served as the negative control.

### Glomerulosclerosis Index

Glomerulosclerosis was assessed using a semi-quantitative technique in a blinded fashion as described previously [27]. In brief, the degree of sclerosis in each glomerulus was subjectively graded on a scale of 0–4: Grade 0, normal; Grade 1, sclerotic area up to 25% (minimal); Grade 2, sclerotic area 25–50% (moderate); Grade 3, sclerotic area 50–75% (moderate to severe) and Grade 4, sclerotic area 75–100% (severe). Glomerulosclerosis was defined as glomerular basement membrane thickening, mesangial hypertrophy and capillary occlusion. A glomerulosclerotic index (GSI) was then calculated using the formula:
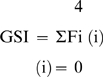



Where Fi is the percentage of glomeruli in the rat with a given score (i).

### Endothelial Cell Density

Glomerular capillary density was determined on JG-12 labelled kidney sections by computer-assisted image analysis (AIS; Analytical Imaging Station version 6.0, St. Catherines, ON, Canada). The percentage glomerular area positive for JG-12 immunostaining was determined in 30 randomly selected glomeruli from each rat in a blinded fashion as previously described [50].

### Cardiac Matrix Deposition

The accumulation of matrix was quantified on picrosirius red stained heart sections using computer-assisted image analysis in a blinded fashion, as previously reported [54]. Briefly, 5 random non-overlapping fields from 6 animals per group were captured from the subendocardium and digitized using a BX50 microscope attached to a Fujix HC5000 digital camera. Digital images were then loaded onto a Pentium III IBM computer. An area of red on picrosirius red sections (for matrix) was selected for their color ranges. Calculation of the proportional area stained was then determined using image analysis, as described [54].

### Myocyte Hypertrophy

The extent of cardiac myocyte hypertrophy, as measured by cross sectional area, was determined on haematoxylin-eosin stained sections in a blinded fashion, as previously published [55].

### Fluorescence Microangiography

Renal (glomerular and tubulointerstitial) microvascular architecture was assessed 8 weeks post-surgery using a slightly modified fluorescence microangiography technique [56]. In brief, animals were anesthetized with inhaled isoflurane 2.5%. The right internal jugular vein was cannulated with a PE50 catheter and 100 U of unfractionated heparin was infused for systemic anticoagulation. The abdominal aorta was cannulated with a 20G plastic catheter with the tip advanced to a point just distal to the renal artery origins. Whole body perfusion-exsanguination commenced at systolic blood pressure (SNX animals: 170–220 mm Hg, sham animals: 120 mm Hg) via the abdominal aorta with PBS (pH 7.4, 100 mL) to remove circulating blood, and the right internal jugular vein was simultaneously severed, allowing free flow of perfusate. The aorta proximal and distal to the renal artery origins was then clamped such that kidney perfusion was isolated. 5 ml of a pre-warmed (50°C) agarose (1%)–fluorescent microbead (10%) (0.02 µm diameter, Invitrogen) mixture was then infused via the 20G catheter into the aorta. After microbead infusion, the animal was cooled with ice for 10 mins, and then the kidney was harvested and immersed in neutral buffered formalin for 24 hrs at 4°C. 200 µm sections were then viewed using a confocal microscope at 20x optical zoom (Leica TCS SL, Leica, Richmond Hill, CA). Neurolucida software (MBF Bioscience, Willitson, VT) was used to digitally reconstruct glomerular and peritubular microvasculature.

### CMTMR Labelling and CMC Tracking

CMCs cultured for 10 days as described were incubated with a 5 µM solution of 5-(and-6)-4-chloromethyl-benzoyl-amino-tetramethylrhodamine (CMTMR, Invitrogen, Carlsbad, CA) for 30 min. 10^6^ CMTMR- cells were infused via tail vein injection into SNX rats 4 weeks after surgery and animals were sacrificed at the time points described. The presence of CMTMR positive cells was determined on a Zeiss LSM510-META confocal microscope by counting CMTMR positive cells in 10 randomly selected 40× magnification fields in a 40 µm frozen section embedded in cryostat matrix (Tissue-Tek, Sakura, Kobe, Japan) [57].

### Statistical Analysis

All data are shown as mean ± SEM unless otherwise stated. A minimum number of 3 independent experiments were performed for all *in vitro* experiments. Urine protein excretion is presented as geometric mean ×/÷ tolerance factors. Differences between groups were analyzed by ANOVA with post-hoc Fisher's Least Significant Difference. All statistics were performed using GraphPad Prism 4.00 for Windows (GraphPad Software, San Diego, CA) or SPSS 15.0 for Windows (SPSS, Chicago, IL). A change was considered statistically significant if *P*<0.05.

## Supporting Information

Figure S1 Intravenous and intra-arterial CMC therapy resulted in similar renal and cardiac protection. (a) Proteinuria. (b) Systolic blood pressure. (c) Glomerulosclerosis. (d) Glomerular endothelial (JG-12) immunostaining. (e) Tubulointerstitial type IV collagen immunostaining. (f) Left ventricular end diastolic pressure-volume relationship. (g) Myocyte cross-sectional area. (h) Cardiac interstitial fibrosis. Abbreviations: SNX - iv: 5/6 nephrectomy (SNX) animal treated with intravenous CMC infusion. SNX - ia: SNX animal treated with intra-arterial CMC infusion. BP: blood pressure. LV EDPVR: left ventricular end diastolic pressure-volume relationship. PSR: picrosirius red.
(0.38 MB TIF)

Figure S2 Renal and cardiac parameters 8 weeks post-surgery in SNX and SNX - SC animals. (a) Tubulointerstitial type IV collagen immunostaining. (b) Glomerulosclerosis index. (c) Left ventricular end diastolic pressure-volume relationship. (d) Cardiac interstitial fibrosis. (e) Myocyte cross-sectional area. Abbreviations: LV EDPVR: left ventricular end diastolic pressure-volume relationship. PSR: picrosirius red. SNX: 5/6 subtotal nephrectomy animal treated with phosphate-buffered saline. SNX - SC: SNX animal treated with bone marrow-derived stromal cells.
(11.17 MB TIF)

Table S1 (0.05 MB DOC)
