# Supplementary figures and images for: Culture-Modified Bone Marrow Cells Attenuate Cardiac and Renal Injury in a Chronic Kidney Disease Rat Model via a Novel Antifibrotic Mechanism
Source: PLoS One. 2010 Mar 4;5(3):e9543. doi: 10.1371/journal.pone.0009543 (PMC2832011; doi:10.1371/journal.pone.0009543)

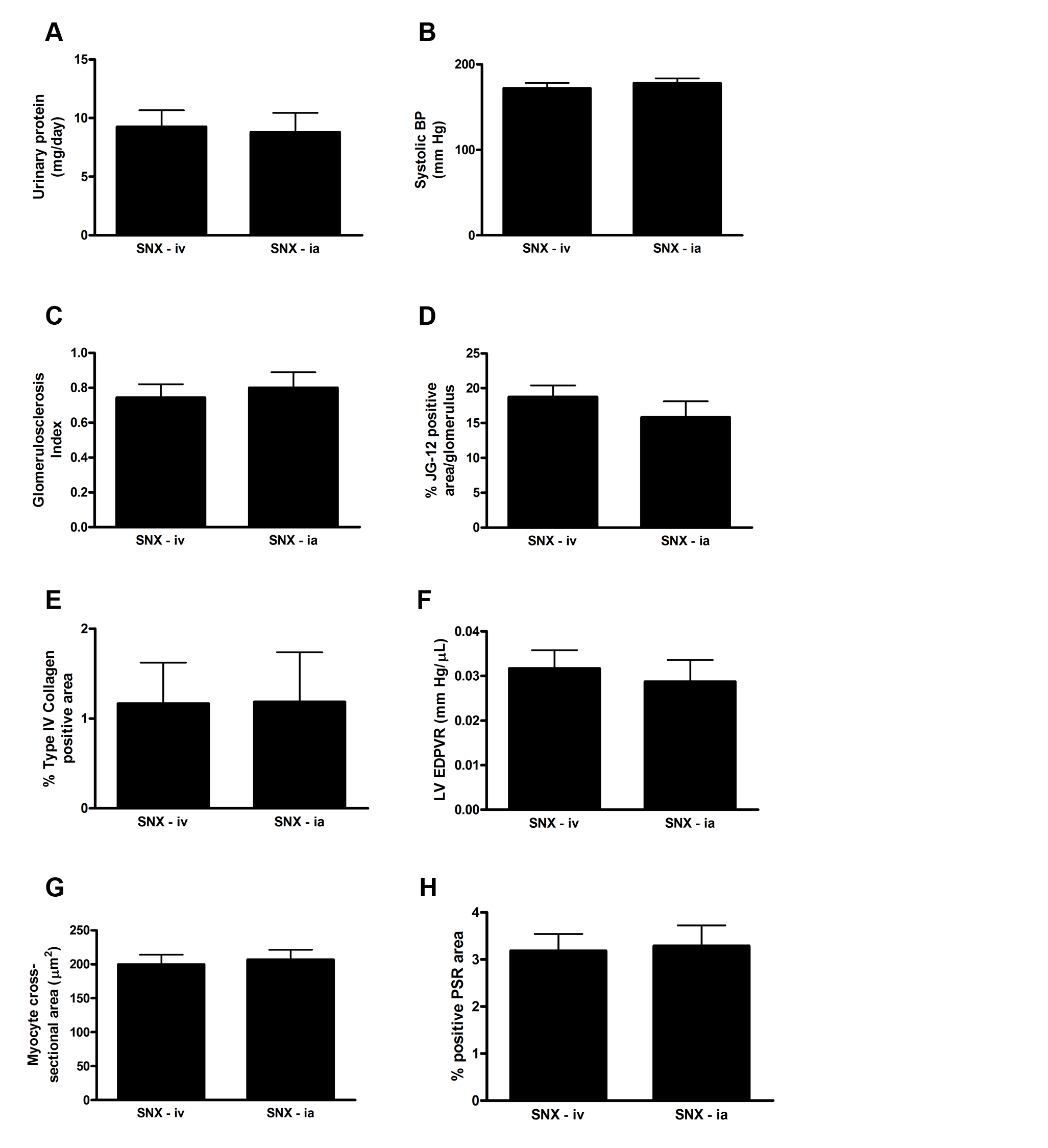

Supplement: Figure S1 — Intravenous and intra-arterial CMC therapy resulted in similar renal and cardiac protection. (a) Proteinuria. (b) Systolic blood pressure. (c) Glomerulosclerosis. (d) Glomerular endothelial (JG-12) immunostaining. (e) Tubulointerstitial type IV collagen immunostaining. (f) Left ventricular end diastolic pressure-volume relationship. (g) Myocyte cross-sectional area. (h) Cardiac interstitial fibrosis. Abbreviations: SNX - iv: 5/6 nephrectomy (SNX) animal treated with intravenous CMC infusion. SNX - ia: SNX animal treated with intra-arterial CMC infusion. BP: blood pressure. LV EDPVR: left ventricular end diastolic pressure-volume relationship. PSR: picrosirius red. (0.38 MB TIF) [file pone.0009543.s001.tif]

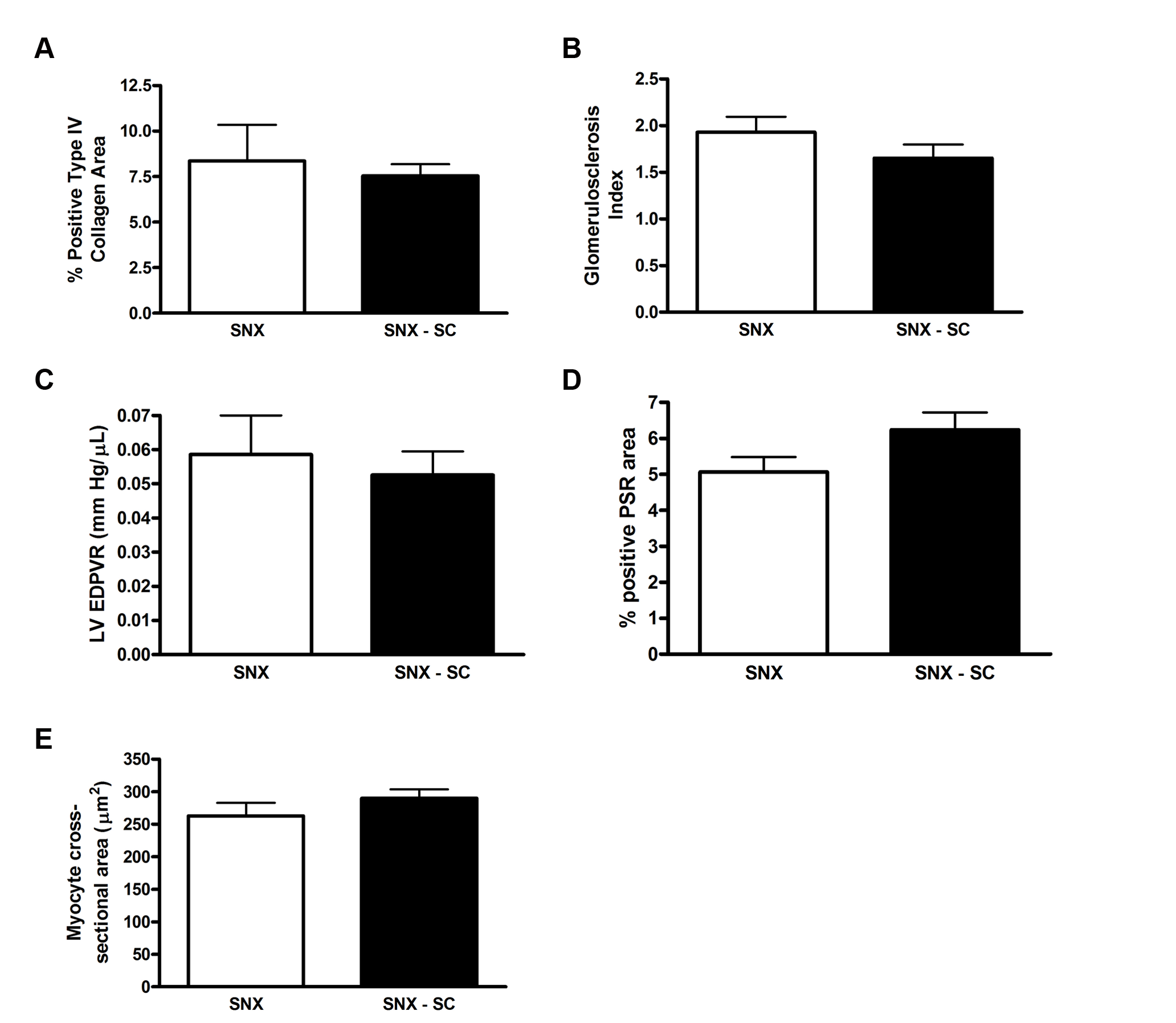

Supplement: Figure S2 — Renal and cardiac parameters 8 weeks post-surgery in SNX and SNX - SC animals. (a) Tubulointerstitial type IV collagen immunostaining. (b) Glomerulosclerosis index. (c) Left ventricular end diastolic pressure-volume relationship. (d) Cardiac interstitial fibrosis. (e) Myocyte cross-sectional area. Abbreviations: LV EDPVR: left ventricular end diastolic pressure-volume relationship. PSR: picrosirius red. SNX: 5/6 subtotal nephrectomy animal treated with phosphate-buffered saline. SNX - SC: SNX animal treated with bone marrow-derived stromal cells. (11.17 MB TIF) [file pone.0009543.s002.tif]
