# Supplementary material for: Culture-Modified Bone Marrow Cells Attenuate Cardiac and Renal Injury in a Chronic Kidney Disease Rat Model via a Novel Antifibrotic Mechanism
Source: PLoS One. 2010 Mar 4;5(3):e9543. doi: 10.1371/journal.pone.0009543 (PMC2832011; doi:10.1371/journal.pone.0009543)
Supplement: Table S1 — (0.05 MB DOC) [file pone.0009543.s003.doc]

**Supplementary Table S1**

|  | **SNX** | **SNX – SC** |
| --- | --- | --- |
| N | 15 | 11 |
| Urine protein (mg/day) | 64.0 x/÷ 1.4 | 49.7 x/÷ 1.2 |
| Plasma creatinine (μmol/L) | 81 ± 11 | 74 ± 9 |
| Systolic BP (mm Hg) | 184 ± 10 | 183 ± 11 |

Urine protein excretion is presented as geometric mean x/÷ tolerance factors. Abbreviations: SNX: SNX animal treated with phosphate buffered saline. SNX – SC: SNX animal treated with bone marrow-derived stromal cells.
